# The Medical Profession

**Published:** 1895-06-22

**Authors:** W. M. Sinclair

**Affiliations:** Archdeacon of London


### The Medical Profession.

By the Ven. W. M. Sinclair, D.D., Archdeacon of London.

[In the course of his sermon at St. Paul's Cathedral on Hospital Sunday, June 16th, 1895, from the text "Himself took our infirmities and bare our sicknness," Dr. Sinclair, preaching on "Christ the Healer," paid an eloquent tribute to the medical profession. Dr. Sinclair's sermon was powerful and manly, and the quotations from our Special Supplement last week were well selected and forcibly put, and the whole of the vast congregation were visibly impressed. The collections, too, were, we hear, nearly 50 per cent. better than in former years.]

He said there is no art or calling more characteristic of our Lord, or more congenial to His will, than that of the healer. When animated by His spirit, there is none that is nobler, or that more richly deserves the reverence and gratitude of mankind, It is a profession that cannot be perfected without long and earnest study, constant practice, accumulated experience, unflinching exposure to danger, and great self-denial. The fact of being so constantly in the presence of anguish and death gives seriousness and gentleness even to the roughest nature. What heroism is shown, not only on great and notable occasions, but even in the most obscure practice, and by young beginners, in the grim fight with death which they so often wage single-handed! What story in that beautiful book of Scottish humour and pathos that has lately appeared is more stimulating or more pathetic than that of the chivalry of the old Forfarshire surgeon amongst the simple peasants of his district? And the rawest student would risk his life by sucking the poison from a desperate throat; no thought of danger stints the efforts of these men in wrestling with the deadly grasp of fever or of small-pox; no weariness of fatigue or yearning for repose prevents the country doctor from starting forth over bleak moor or mountain pass, in snow or rain or any weather, in the dead of night, or when he has returned from his day's round, if he be summoned by the call of pain and sudden disease. When our friends are ill, how implicitly we trust the doctor's experience, how eagerly we look for his visit, with what trembling anxiety we listen to his verdict, how we bless his hands when beyond expectation, under God's good providence, some alleviation is wrought, and a change comes for the better, and gradually strength returns. Or even when death is inevitable, how often the sufferings of the last days and hours are mitigated by medical attention and experience!

Of the miracles of the surgical art it is impossible here to speak in detail. It is little to say that operations are now performed which would once have seemed incredible as fairy tales. Enviable indeed must be the feeling of the brilliant man of science as, with calm eye, keen instinct, unerring knowledge, and unswerving hand, he removes some horrible evil, or puts to right some terrible complication, which would otherwise have brought life to an abrupt and hideous close. I was a few months ago dining in the very room in which Sir James Simpson, the inventor of chloroform, had the courage to experiment on himself for the first time. Few rooms have been more rich in blessing to mankind than that. Think merely of what happened after a battlefield in days gone by, when limbs were hurriedly sawn off amidst shrieks and groans as frightful as the cries of the field of slaughter itself. And now the wounded soldier goes peacefully off to sleep, and, dreaming of home and mother, wakes to find his trouble all removed, and all the pain and suffering over!

It is not because we ourselves are cowardly, or refuse to die, or to take our share of suffering, that we honour our physicians and surgeons, and repay them with affection and gratitude, but it is because we love our friends whose lives they preserve to us, because it is so often the bread-winner whom God enables them to rescue, because very often ruin or lifelong and incurable grief would be the result if they were not at hand to baffle the enemy. When we think of a young wife and little children rescued from want, or the treasure of the heart saved, or a life valuable to a whole community prolonged, and contrast with these pictures that of the home wrecked and desolate, the light of the eyes darkened, hopes dashed to the ground, prospects blighted for ever, lives maimed, crippled, and rendered useless, then indeed we do not wonder that the cause of the healing of sickness was so dear to the heart of our Lord, and we hail in benevolent physician and skilful surgeon the sons of Him who Himself took our infirmities and bare our sicknesses.

Would that the congregation of St. Paul's Cathedral, who are so very rarely asked to contribute to anything whatsoever, would take this opportunity of showing their gratitude to God for all the spiritual privileges which they here enjoy! Would that this day was treasured and anticipated amongst you as a great annual festival of charity and national benevolence! Would that this cathedral, the cathedral of the metropolis of the whole Empire, might lead the way! Might not the illustrious Colleges of Physicians and of Surgeons, and the governors of the great hospitals, and the representatives of the great nursing institutions, whether they consider Hospital Sunday a helpful idea or not, use this morning's service as an occasion for publicly acknowledging to Almighty God the wonderful blessing that He has bestowed on their beneficent labours during the previous twelve months!

